# Assessing the congruence between perceived connectivity and network centrality measures specific to pandemic influenza preparedness in Alberta

**DOI:** 10.1186/1471-2458-10-124

**Published:** 2010-03-10

**Authors:** Justin N Hall, Spencer Moore, Alan Shiell

**Affiliations:** 1 School of Kinesiology & Health Studies, Queen's University, Kingston, Canada; 2 Population Health Intervention Research Centre, Department of Community Health Sciences, University of Calgary, Calgary, Canada

## Abstract

**Background:**

Recent research has suggested that perceived organizational connectivity may serve as an important measure of public health preparedness. Presumably, organizations with higher perceived connectivity also have a greater number of actual organizational ties. Using network analysis, we evaluate this presumption by assessing the correlation between perceived organizational connectivity and reported inter-organizational connections.

**Methods:**

During late 2007-early 2008, representatives from organizations involved in the delivery of public health systems in Alberta were asked to complete an online questionnaire on public health preparedness. Organizational jurisdictional information was collected. Items from Dorn and colleagues connectivity scale (2007) were used to measure perceived organizational connectivity. Inter-organizational network data on formal connections in the area of pandemic influenza preparedness were collected using a roster approach. These data were imported into UCINET to calculate in- and out-degree centrality scores for each organization. One-way ANOVA tests assessed if perceived connectivity and in- and out-degree centrality varied among jurisdictions. Pearson correlation coefficients were used to assess the correlation of perceived connectivity and in- and out-degree centrality.

**Results:**

Significant mean differences among jurisdictions were observed for in-degree (*F*(3,116) = 26.60, *p *< 0.001) and between provincial and lower jurisdictions for out-degree centrality (*F*(3,116) = 5.24, *p *< 0.01). Higher jurisdictions had higher average centrality. Perceived organizational connectivity was correlated with out-degree (*r*(123) = 0.22, *p *< 0.05) but not in-degree centrality (*r*(123) = -0.07, *p *> 0.05).

**Conclusions:**

The results suggest in terms of pandemic preparedness that perceived connectivity may serve as a partial proxy measure of formal out-degree network connectivity.

## Background

In a public health emergency, an immediate response is required to mitigate the extent of injury, disease, disability, and death suffered by the population. It is imperative that agencies with overlapping responsibilities coalesce quickly, provide care to those affected, implement control measures to minimize the scope of the emergency, keep critical community services and businesses operational during the emergency, and restore health and community services back to normal operation[1]. Since the timing and location of most emergencies are not predictable, agencies may not have time to prepare during the midst of a crisis, rather they must leverage information, assets, and response capacity, and coordinate response initiatives to the current situation almost immediately[2]. Being connected to other organizations is a critical element in the capacity of agencies to access and mobilize resources within the public health preparedness network.

Connectivity has been defined as a "seamless web of people, organizations, resources, and information that can best catch, contain, and recover from a terrorist incident or other disaster."[3] The concept has been described as useful for describing the integration and coordination of the public health and emergency management network[3]. It has been reported that even if a system has ample resources in terms of equipment, training, and funding, these components will not function optimally if the system does not reach a threshold level of connectivity[4]. If, however, organizations have developed strong and mutual relationships, research suggests that the system as a whole will likely manage the crisis more efficiently and have greater capacity to adapt and improvise should an unexpected event outside of the specific action and contingency plans occur[3,4].

Recent research on organizational connectivity and public health preparedness has suggested that perceived organizational connectivity is a useful proxy indicator of organizational preparedness[2]. Presumably, this is because perceived connectivity indicates actual organizational connectivity. Indeed, in terms of accessing information and resources through the system, what matters during an emergency is not whether organizations perceive themselves as connected but whether they actually have those connections in place. In this study, we address whether perceived organizational connectivity serves as a proxy measure of formal ties objectively reported or received by organizations with respect to pandemic influenza preparedness. It is hypothesized that perceived and network connectivity are correlated.

Of special interest about this study is the use of sociometric network data from over 100 public health and emergency management agencies to assess inter-organizational network connections. Inter-organizational networks consist of the relational ties among organizations and the overall structure resulting from those interconnections[5]. An inter-organizational network approach reveals the degree of integration or fragmentation of service delivery existing among organizations[6]. Integration refers to the extent of interconnectedness among organizations, and service delivery outcomes are often shown improved with greater integration[7,8]. Network analysis can also provide insight into which organizations are more influential or prominent in the overall system. Central actors are seen as those actors that are more involved or prominent in the network. This form of centrality is not the result of organizational perceptions. Instead, it emerges from how the various organizations identify the types of connections they have to other specific organizations. In this regard, centrality can be distinguished between in-degree and out-degree centrality. In-degree centrality measures the connections that an organization receives from others within the network while out-degree centrality measures the connections that an organization sends to or reports having to other agencies within the network.

Given the greater costs and higher respondent burden associated with administering and completing an inter-organizational network questionnaire, information gleaned from this analysis could be invaluable for the development of questionnaires that provide similar information but are less costly and have a lower respondent burden. If we can show that perceived connectivity parallels actual network connectivity, then the results of this work could offer an effective and timely means of assessing organizational connectivity, a critical component of public health preparedness, without the need for formal network analysis.

## Methods

### Sample description

The current work used data collected through the "Public Health Preparedness and Responsiveness in Alberta: An Inter-Organizational Relations Study of Public Health Preparedness and Response Networks" research project. Using a stratified random sampling technique, 595 organizations geographically spread across four jurisdictional levels (provincial, regional, city, and town/village) were invited to complete an online questionnaire. In an initial census, these organizations were identified as potentially playing a role in public health preparedness or emergency management within the province. Email and regular mail invitations were sent to organizational representatives who were asked to select the most appropriate person in their organization to complete the online questionnaire.

### Organizational questionnaire

The online organizational questionnaire was developed specifically for this study to assess public health preparedness in Alberta (see Additional file 1). Questionnaires were completed between November 2007 and April 2008. The questionnaire asked organizational representatives about the general characteristics of organizations, subjective assessments of the institutional environment, and general perceptions of their system and organizational connectivity, and inter-organizational network relations. A roster approach, in which organizations were provided with a listing of organizations with which they may have relations, was used to collect sociometric network data. To facilitate response ease, the listing of 595 organizations was first divided into jurisdictional and geographical areas. All respondents were first asked if they had ties to organizations at provincial, sub-provincial, and city levels. Respondents were then asked if they had formal ties to any organizations within separate town/village areas grouped according to geographical area. If they responded yes, they were presented the listing of organizations within the town/village areas that they selected. Representatives identified those organizations at each level with which their own organization had ties in terms of formal and informal relationships and information sharing in relation to pandemic influenza. Formal ties were those in which written responsibilities and obligations had been developed. Informal ties were those in which communication occurred between agencies without official responsibilities or written obligations.

### Measures

#### Organizational connectivity

Perceived organizational connectivity was measured using organizational connectivity items from the connectivity instrument developed by Dorn and colleagues[2]. Organizational connectivity items asked respondents to assess their level of confidence on a four-point Likert scale from not confident to very confident to 1) perform the tasks that the organization is expected to accomplish, 2) make connections to other organizations for which the organization is responsible, 3) provide assistance and information to others, 4) acquire assistance and information from others, 5) perform cooperative and connected activities with other people and organizations, and 6) manage differences and disputes. A total summative score was obtained for each responding organization.

Organizational network connectivity was measured using the Freeman centrality measure[9]. For non-symmetric data (as is the case in our work), the in-degree of an organization is the number of ties received by the organization and the out-degree is the number of ties initiated by the organization[9,10]. Network data on the formal ties that organizations had to one another with regards to pandemic influenza preparedness were cleaned and imported to UCINET, version 6.211[10]. In-degree and out-degree Freeman centrality scores were calculated for each participating organization.

#### Organizational jurisdiction

Organizational jurisdiction was divided into four strata: 1) provincial-level departments, 2) sub-provincial administrative agencies, i.e., emergency management districts (disaster services) and regional health authorities, 3) cities, and 4) towns and village areas. According to the 2006 Census of Canada, the average population size of Alberta cities, towns, and village areas was 136,613 people, 3,835 people, and 400 people, respectively[11].

### Statistical analysis

First, the internal consistency of the perceived organizational connectivity scale was calculated by means of Cronbach's alpha[12]. A high alpha (0.7 and higher) is consistent with the hypothesis that all of the scale items are measuring the same construct. Second, ANOVA tests were performed to assess if there were significant differences in network centrality scores among the four jurisdictional levels. Third, Pearson correlation coefficients were computed between network centrality and perceived organizational connectivity scores for the entire sample. Statistical analyses were performed using SPSS, version 16.0.

### Funding and ethics

This project was funded by the Alberta Heritage Foundation for Medical Research and received ethics approval from the Office of Biomedical Ethics of the Faculty of Medicine, University of Calgary and from the Health Research Ethics Board of the Faculty of Medicine and Dentistry, University of Alberta.

## Results

### Response rates and demographics

**Figure 1:**
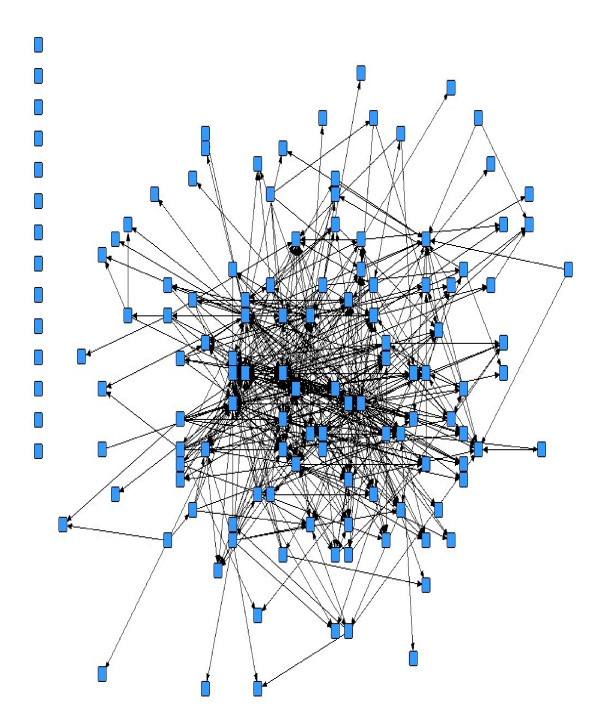
**Formal ties pandemic influenza respondent network (n = 135)**. Names of individual organizations are not provided due to confidentiality agreements associated with the data collection

**Figure 2:**
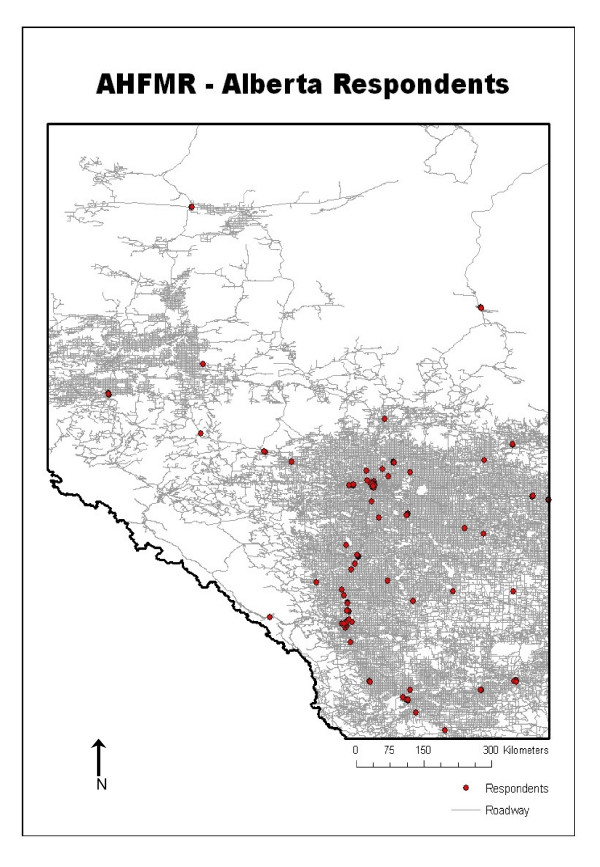
Geographical distribution of organizational survey respondents throughout Alberta

Of the 595 organizations invited to complete the organizational questionnaire, a total of 135 organizational representatives responded to the network assessment component (Figure 1). Of those 135, 10 additional organizations had to be dropped from this analysis since they did not provide perceived organizational connectivity information. Several departments chose to respond as part of a larger organization or agency within a particular city or local region. At the town level, many organizations that were asked to participate did not see their organization's mission as concerning public health preparedness and chose not to respond. In terms of respondent coverage, the response percentage at each jurisdictional level was as follows: provincial (45.2%), regional (37.9%), city (30.7%), and town/village (20.9%). Despite the low overall organizational-level response rate, respondent data are geographically and jurisdictionally spread across agencies involved in public health preparedness. For example, in terms of geographical sample coverage, 87.5% of Alberta cities and 69.1% of the towns and villages sampled provided at least one organizational respondent (Figure 2). In 68.8% of the cases at the city level and 59.5% of the cases at the town/village level, respondents represented fire department or disaster services organizations. Descriptive statistics outlining perceived organizational connectivity score, network connectivity scores (in-degree and out-degree centrality), and scores stratified by jurisdictional level are reported in Table 1.

**Table 1:** Alberta public health preparedness sample: Descriptive statistics (n = 125)

Variable	Mean (Standard Deviation)
*Perceived Organizational Connectivity Score*	16.88 (3.92)
	
*Perceived Organizational Connectivity Score by Jurisdiction*	
- Provincial	16.81 (3.80)
- Regional	17.32 (4.20)
- City	17.70 (3.77)
- Town/Village	16.16 (3.79)
	
*Network Connectivity Scores*	
- In-Degree Centrality	4.32 (5.54)
- Out-Degree Centrality	4.32 (7.65)
	
*In-Degree Centrality by Jurisdiction*†	
- Provincial	12.26 (8.20)
- Regional	5.06 (2.39)
- City	3.48 (2.37)
- Town/Village	1.71 (0.94)
	
Out-Degree Centrality by Jurisdiction*	
- Provincial	16.31 (13.52)
- Regional	5.07 (6.12)
- City	6.33 (5.45)
- Town/Village	6.04 (5.12)

† Significant mean differences among all jurisdiction levels (ANOVA tests)

* Significant mean difference between provincial and lower jurisdictional levels only (ANOVA tests)

### Perceived organizational connectivity scale

The Cronbach's alpha coefficient for the perceived connectivity scale was 0.93, showing high internal consistency among the items. All items were positively correlated to each other and the correlation coefficients between the items ranged from 0.57 to 0.82.

### ANOVA tests

Three separate one-way between-subjects analysis of variance (ANOVA) tests were conducted. The first ANOVA test comparing perceived connectivity scores across four jurisdictional levels showed that perceived organizational connectivity did not differ significantly among levels. The second ANOVA test examining differences in in-degree centrality scores across jurisdictional levels found that in-degree centrality did vary significantly among levels (*F*(3,116) = 26.60, *p *< 0.001). The third ANOVA test comparing out-degree centrality scores across jurisdictional levels also found out-degree to vary significantly (*F*(3,116) = 5.24, *p *< 0.01) with differences existing solely between provincial and all the lower levels.

### Correlation analysis

A Pearson correlation matrix of perceived connectivity score and network connectivity scores (in- and out-degree centralities) is displayed in Table 2. Perceived connectivity was significantly correlated with out-degree centrality (*r*(123) = 0.22, *p *< 0.05), but not with in-degree centrality (*r*(123) = -0.7, *p *> 0.05). In-degree and out-degree centralities were also significantly correlated with each other (*r*(123) = 0.24, *p *< 0.01).

**Table 2:** Pearson correlation matrix of perceived connectivity score, in-degree centrality score, and out-degree centrality score

	Perceived Connectivity	In-Degree Centrality
**In-Degree Centrality**	-0.07	---

**Out-Degree Centrality**	0.22*	0.24**

*p < 0.05

**p < 0.01

## Discussion

Perceived organizational connectivity was correlated with out-degree network centrality but not in-degree centrality. These results suggest that perceived organizational connectivity may serve as a partial proxy measure of formal ties objectively reported, but not received, by organizations with respect to pandemic influenza preparedness. This finding makes intuitive sense since ties that other organizations report to an organization of interest may not be recognized or reciprocated. This may explain why in-degree centrality is not statistically associated with organizational connectivity perceptions. Out-degree centrality may be biased by organization's perceptions of their own influence in a way in which in-degree scores are not.

Further understanding of these results can be gained through consideration of the concept of cognitive accuracy. Krackhardt (1990) defined cognitive accuracy as the degree to which a person's perceived networks correspond to actual networks[13]. His work concluded that an actor's accuracy in perceiving the influence of other actors in the network was positively associated with actual influence in the network. Choi and Brower (2006) extended the concept of cognitive accuracy to collective cognitive accuracy in their research assessing local emergency management systems in Florida[14]. Collective cognitive accuracy shows how well participants are aware of the networks in practice and which participants are influential within each network. It is calculated as a simple percentage of the responding organizations that accurately identify the influence of an organization within a network (as compared to the relationships identified through a formal network analysis)[14]. Choi and Brower's work supported the conclusion that collective cognitive accuracy has a strong positive relationship with the centrality of networks, and that when actors have a clear mental picture of the network, greater collaboration and information sharing is observed, resulting in more effective decision making and actions[14]. Unlike the current results however, these authors noted a strong correlation with in-degree centrality. One possible explanation for this discrepancy is that the networks in Choi and Brower's work were decentralized horizontal networks with distributed power and authority structures, rather than the primarily hierarchically-linked organizations that were the subject of our work. The scope of the network in Choi and Brower's work was focused at the local level only while our work had a much larger network structure, focused at multiple jurisdictional levels. Furthermore, Carley, Lee, and Krackhardt (2001) have previously shown that distributed decentralized networks exhibit dynamic patterns which are very different than those found in hierarchical networks[15]. These differences in structural dynamics may offer an explanation as to why in-degree centrality was significantly correlated with organizational connectivity in our work, rather than out-degree centrality. Further research is needed to examine these inter-organizational relationships in greater detail.

Using an organizational network analysis approach provides insight into the overall structure and types of relationships existing in the public health preparedness system in Alberta for pandemic influenza. This work helps address a general gap in the public health field related to organizational network analysis, identified by Luke and Harris (2007) in their work on network analysis methods and applications in public health, in that it helps provide a structural evaluation of public health systems[16]. With the recent shift and emphasis on using a systems approach to design, study, and evaluate public health programs,[16] the findings of this study offer a technical means of assessing the extent to which each organization in a network is linked to others and the patterns of relationships among different organizations for one specific case example on pandemic influenza. Not only do these findings provide a means of illustrating how organizations function together as a unit, but it serves to offer an effective and timely means of assessing organizational connectivity, a critical component of public health preparedness. Moreover, formally identifying organizational connections using out-degree centrality scores or using the partial proxy measure of perceived connectivity provides a valuable tool for identifying the prominence of organizations in a network. This information can aid policymakers in developing strategies for collaboration that build on the current strengths of highly connected and central organizations while enhancing the capacity of less connected and more peripheral organizations[7,16,17].

Analysis of variance tests revealed statistically significant differences in in-degree and out-degree centrality measures by jurisdictional level. Differences were observed among all jurisdictional level combinations for in-degree centrality as well as between provincial and each of regional, city, and town/village levels with respect to out-degree centrality. In each case, the higher jurisdictional level had a significantly higher centrality score indicating these organizations were more connected to surrounding organizations. This finding would seem to confirm the hierarchical character of the preparedness system in Alberta as organizations at higher jurisdictions receive and report greater formal connections.

Limited literature exists which assesses the role of jurisdiction on centrality, connectivity, coordination, or specific preparedness measures for public health organizations, making it challenging to provide support for the current results. Also, unique to the current work is the depth of coverage of the inter-organizational network for pandemic influenza at four jurisdictional levels. Most other studies have typically compared only two levels, for example local and state, or county and regional levels[18,19]. In general, however, our results are supported by recent work in Florida examining emergency management networks. According to this work, smaller municipalities have primarily relied upon counties or regional network supports for disaster-related functions since higher jurisdictional levels are better prepared for managing emergencies[20]. Networking is advantageous because links form between individuals and agencies that would work together during a public health emergency and this serves to heighten capacity and address metropolitan fragmentation issues which are often present at lower jurisdictional levels[18]. Other U.S. research has indicated that aside from major metropolitan areas, few counties, cities, or towns have the capacity to respond to public health emergencies independently since they lack the necessary coordination or supports with other agencies[18]. The greater interconnectedness observed at higher jurisdictional levels in the current work is important as it may enhance communication, likely decreases coordination problems during the time of an emergency, and builds cohesion that could enhance preparedness.

There are several limitations of this work worth considering. First, the study compares perceived connectivity with network centrality arising from an organization's formal ties and relationships. Although we found out-degree centrality based on formal ties correlated with perceived connectivity, it is conceivable that other dimensions of inter-organizational relationships, such as information or material-resource sharing, may or may not be correlated with perceived connectivity in the same fashion. Further research is needed to examine if perceived connectivity is more closely linked to other dimensions of inter-organizational relationships. Second, this study is of one specific provincial preparedness network. Since the conclusion of data collection for this project, Alberta has reorganized its public health delivery system, particularly at the regional level. As a result, the type of network connections described in this study may not be the same as those currently in place. Finally, this is a cross-sectional analysis and the network data are from one moment in time. Yet, the networks in which organizations are embedded are dynamic[15]. With recent emphases on preparedness planning, the development of action plans, and the increased use of simulation exercises, networks are continually evolving to reflect the changing landscape. The Alberta sample is no exception, and while data is collected from one time period there remains value in studying the structure and composition of the Alberta preparedness network as it existed in late 2007-early 2008. This makes possible in part the further monitoring of how relationships form and become institutionalized[14]. Future research can use such information as a starting point for designing network- or system-level surveillance systems of organizational preparedness and conduct more dynamic analyses using longitudinal data on the structure of preparedness and response networks. While this study was conducted prior to the current H1N1 outbreak, examining how this recent event has tested the public health system and the connectivity within it would be a logical next step as investigators would likely find today a very different connectivity and set of patterns in the field.

## Conclusions

The current work assessed the relationship between perceptions of organizational connectivity and the objective assessment of connectivity revealed through network analysis and centrality measures. First, correlation analysis results suggested that perceived organizational connectivity serves as a partial proxy measure of formal ties objectively reported, but not received, by organizations with respect to pandemic influenza preparedness in Alberta. Whether perceived connectivity and its network correlate of out-degree centrality provide a more valid indicator of preparedness than in-degree centrality, for example, remains a subject for future research. A practical application stemming from this study's findings is that organizations involved in preparedness activities might use perceived connectivity as an efficient, effective, and timely measure of organizational connectivity. These measures can be collected through a brief organizational survey which asks six questions related to perceptions of connectivity rather than performing a formal network analysis. This is an invaluable exercise since identifying possible gaps in how organizations feel connected to the system through a brief survey will possibly allow more time to address those gaps before they become a burden during an emergency. Second, significant differences in centrality scores were observed by jurisdictional level with higher jurisdictional organizations displaying greater degrees of connectedness on average. Since lower jurisdictional agencies appear less well connected in the system, this information can be used to build more effective community-based coalitions. In other words, these organizations should be targeted for programs that develop and foster their connections with other surrounding organizations. Improving formal ties should allow for greater collaboration, open communication, teamwork, and heightened overall response capacity and better allow the system to respond with flexibility and resilience during a public health emergency.

## Competing interests

The authors declare that they have no competing interests.

## Authors' contributions

JNH originated the study, and led the writing and analyses. SM assisted in the study and statistical analyses. AS assisted in the study and analyses. All authors helped to conceptualize ideas, interpret findings, and review drafts of the article. All authors read and approved the final version of the manuscript to be published.

## Pre-publication history

The pre-publication history for this paper can be accessed here:

http://www.biomedcentral.com/1471-2458/10/124/prepub

## Supplementary Material

Additional file 1 **Alberta public health preparedness organizational survey**. BMCPH  Organizational survey Click here for file
