# Supplementary material for: Assessing the congruence between perceived connectivity and network centrality measures specific to pandemic influenza preparedness in Alberta
Source: BMC Public Health. 2010 Mar 10;10:124. doi: 10.1186/1471-2458-10-124 (PMC2841105; doi:10.1186/1471-2458-10-124)
Supplement: Additional file 1 — Alberta public health preparedness organizational survey. BMCPH Organizational survey [file 1471-2458-10-124-S1.PDF]

ORGANIZATIONAL ID#: __________
Organizational Name: ________________________________

ALBERTA PUBLIC HEALTH PREPAREDNESS
ORGANIZATIONAL SURVEY

I. ORGANIZATIONAL ATTRIBUTES

A. General Organizational Demographics

1. Please indicate the name of your organization:
   ____

2. When was your organization established? _________

3. Please indicate your job title inside your organization:
   ____

4. What is (are) the principal area(s) of focus of your organization?

   _______ Emergency Management (EM)
   _______ Public Health Preparedness (PHP)
   _______ Both
   _______ Other (Please describe: _____________________)

5. What would you consider the scope of jurisdiction of your organization's emergency management/public health preparedness activities or programs to be:
   (check all that apply)

   ______ town/village (local)
   ______ city
   ______ sub-provincial (e.g., Regional Health Authority or Emergency Management District)
   ______ provincial (covers the entire province of Alberta)

6. As of January 1, 2006, how many employees worked on PHP/EM activities in your organization? (Please include part-time employees as half-time, e.g., 1.0 for full-time and 0.5 for part-time employee)
   ____

7. As of January 1, 2006, how many volunteers worked on PHP/EM activities in your organization?
   ____

8. Please indicate the total budget (including equipment, overhead, support staff, management) allocated to emergency management and/or public health preparedness activities and programs inside your organization during 2006:
   ____

B. General Organizational Employee Training Questions

9. To familiarize workers with various emergency roles, which opportunities does your agency provide? (check all that apply)
   9a) _____ Training among your own staff
   9b) _____ Joint Training among your staff and other community response personnel
   9c) _____ Your Agency provides other opportunities
   9d) _____ No training opportunities

	Question	Yes	No	Don't Know (DK)	NA
10.	Does your agency conduct an internal training needs assessment to identify gaps in employee knowledge, skills, and abilities?				
11.	Do agency employees participate in training exercises or courses on public health preparedness sponsored by Alberta Health and Wellness?				
12.	Do agency employees participate in training exercises or courses on emergency management sponsored by Alberta Municipal Affairs?				
13.	Do agency employees participate in training exercises or courses on emergency management sponsored by Public Health Agency of Canada or another federal agency?				
14.	Does your agency have written job descriptions for PHP employees?				
15.	Does your agency have written job descriptions for EM employees?				
16.	Do these written job descriptions define knowledge, skills, and abilities needed for emergency roles and responsibilities?				
17.	Has your agency adopted job descriptions/skills requirements established by other organizations or in conformity with recognized standards for training and certification?				

C. Organizational Perceptions of the Environment

In this part of the survey you will be asked your opinions on the resources available to your organization (department or office) in support of the performance or development of its PHP/EM activities.

18. Please rate your level of satisfaction with the availability of the following resources to support your organization's activities in PHP/EM:

	Type of Resources Available	Level of Satisfaction			
		Very Dissatisfied	Dissatisfied	Satisfied	Very Satisfied
a)	Availability of personnel trained in public health.				
b)	Availability of personnel trained in emergency management.				
c)	Availability of training programs for staff.				
d)	Availability of technology & equipment for PHP/EM.				
e)	Availability of information on regional activities related to PHP/EM.				
f)	Availability of information on other organizations' PHP/EM activities in your area.				
g)	Availability of information on provincial activities related to PHP/EM.				
h)	Availability of information on provincial sources of funding.				
i)	Overall **provincial** funding level for PHP activities.				
j)	Overall **provincial** funding level for EM activities				

k)	Overall **federal** funding level for PHP activities.				
l)	Overall **federal** funding level for EM activities.				
m)	Overall **provincial** regulatory support for your organization's PHP/EM activities.				
n)	Overall **federal** regulatory support for your organization's PHP/EM activities.				

D. System and Organizational Level Connectivity

For the following section of questions, please indicate the level of confidence that you have ***first of all*** in the **Alberta PHP/EM system as a whole** (i.e., all organizations and departments not only yours) and ***secondly*** in **your own organization's** ability to accomplish or meet the specific functions described below.

19. System Level Connections

	**How confident do you feel in your system's ability to…**	Not Confident	Somewhat Confident	Confident	Very Confident	Don't Know
a)	perform the tasks that the *system* is expected to accomplish.					
b)	make the connections among organizations that are necessary for *system* operation.					
c)	provide information from one organization to another.					
d)	acquire assistance and information from one organization to another.					
e)	perform cooperative and connected activities within the *system.*					
f)	manage differences and disputes.					

20.	**How would you rate the overall…**	Very Poor	Poor	Good	Very Good	Don't Know
a)	connectivity of people and organizations within your system.					
b)	connectivity of your system during an actual emergency to other parts of the preparedness system.					

21. Organizational Connections

	**How confident do you feel in your organization's ability to…**	Not Confident	Somewhat Confident	Confident	Very Confident	Don't Know
a)	perform the tasks that the *organization* is expected to accomplish.					
b)	make the connections to other organizations for which your *organization* is responsible.					
c)	provide assistance and information to others.					
d)	acquire assistance and information from others.					
e)	perform cooperative and connected activities with other people and organizations.					
f)	manage differences and disputes.					

22.	**How would you rate the overall…**	Very Poor	Poor	Good	Very Good	Don't Know
	connectivity of your organization to other parts of the preparedness system.					

II. ORGANIZATIONAL PHP/EM CAPACITY INVENTORIES

23a) Does your organization have a written emergency or public health emergency response plan?

___ Yes
___ No

23b) Has your organization updated this plan since 2004?

___ Yes
___ No

23c) Did your organization exercise this plan either in practice or in a real situation during 2006?

___ Yes
___ No

For the following items related to ***pandemic influenza preparedness and response***, please check ***yes***, ***in-progress***, or ***no***. If you don't think the item is applicable to your organization, select ***NA*** (not applicable). If you are not sure or don't know, select ***DK*** (don't know).

PANDEMIC INFLUENZA CAPACITY CHECKLIST

	**Has your agency or organization…**	Yes	In-Progress	No	NA	DK
24.	**Surveillance**					
a)	established links with avian/swine influenza surveillance contacts within the province?					
b)	developed or improved communication mechanisms for the rapid and timely exchange of surveillance information between province, health region, and local stakeholders?					
25.	**Vaccine Programs**					
a)	increased annual influenza vaccination coverage rates among health care and essential services workers?					
b)	determined the number of people within your agency's jurisdiction who fall within each of the priority groups for vaccination (i.e., high risk groups, health care workers, emergency service workers, specific age groups)?					
26.	**Health Services Emergency Planning**					
a)	developed provincial, regional, or local guidelines for prioritizing health care needs and service delivery or accessing resources and implementing infection control measures during a pandemic?					
b)	assessed health care personnel capacity: estimated the number of HCW (health care workers) by type (physician, nurses, respiratory therapists, etc.) and by work setting (hospital, community) and number of non-active HCW retired?					
c)	determined sources from which additional HCWs and volunteers could be acquired, including Emergency Measures Organizations and NGOs (Red Cross, St. John Ambulance) in pandemic					

	planning?					
d)	determined the number and type of health care facilities and estimated their capacity (e.g., hospital beds, ICU beds, swing beds)?					
e)	determined potential alternative sites for medical care (possible sites could include shelters, schools)?					
f)	determined the capacity of mortuary/burial services, as well as social and psychological services for families of victims?					
g)	coordinated clinical care and health services plans with bordering jurisdictions to avoid migration to centres of perceived enhanced services?					
27.	**Emergency Planning and Response**					
a)	developed contingency plans to provide essential community services such as food, medical and other essential life support needs including plans for emergency back-up of such services?					
b)	estimated the numbers of emergency-services workers including police, fire, correctional, military, funeral services, utilities, telecommunications (e.g., managers of response teams) within your jurisdiction, essential to pandemic response?					
c)	identified voluntary organizations, which would assist during a pandemic?					
d)	conducted assessments of surge capacity of hospitals, alternate care sites, and other facilities?					
e)	developed post-emergency recovery plans?					
28.	**Communications**					
a)	translated public health messages directed to the public into additional languages based on local demographics (e.g., Chinese, Spanish, Russian)?					

b)	identified gaps in the existing communications systems that will require additional resources?					
c)	developed plans and mechanisms for communicating quickly and consistently with other jurisdictions and organizations?					
d)	developed plans and mechanisms for communications with all relevant audiences, including media, key opinion leaders, stakeholders, employees?					
e)	pilot tested and evaluated "single-window" points of contact in involved jurisdictions and organizations to ensure ease of access and ensure names/numbers/e-mails are up-to-date?					
f)	established a primary spokesperson who will speak for the organization on the major events?					
29.	**Public Health Measures**					
a)	established professional and public education and/or strategies for each phase of a pandemic?					
b)	identified staffing needs and staff resource requirements for a pandemic response?					
c)	developed protocols for case and contact management, including the implementation of quarantine and community-based measures?					
d)	developed protocols for school closures and cancelling or restricting public gatherings?					
e)	engaged community stakeholders (e.g., school boards, businesses) in the planning process for community-based control measures?					
f)	assessed how border measures may impact your jurisdiction and inform and plan with stakeholders (e.g., airports) how these measures can be coordinated?					

For the following items related to *West Nile Virus preparedness and response*, please check ***yes, in-progress,* or *no*.** If you don't think the item is applicable to your organization, select **NA** (not applicable). If you are not sure or don't know, select **DK** (don't know

WEST NILE VIRUS CHECKLIST

30.	**Has your agency or organization…**	Yes	In-Progress	No	NA	DK
a)	established a WNV Task Force to foster communication and developed a working relationship among all agencies and individuals potentially involved in WNV response?					
b)	developed a written WNV response plan that identifies roles and responsibilities for monitoring and responding to local WNV activity (ranging from normal season to epidemic conditions)?					
c)	identified specific emergency needs in response to a WNV outbreak?					
d)	established monitoring and communication systems for WNV?					
e)	established communication channels for reporting number of WNV cases on a regular basis?					
f)	prepared a public education program on WNV (e.g., mosquito control, disease symptom knowledge)?					
g)	prepared a public education program on WNV for handling dead birds?					
h)	prepared a public education program on WNV for horse owners?					
i)	identified high risk areas?					
j)	identified high risk populations?					
k)	identified a provincial laboratory for WNV					

	testing?					
l)	developed & maintained a local surveillance network on WNV for birds?					
m)	developed & maintained a local surveillance network on WNV for mosquitoes?					
n)	developed & maintained a local surveillance network on WNV for horses?					
o)	developed & maintained a local surveillance network on WNV for humans?					
p)	made plans to coordinate the pick-up and shipping of dead birds for virus testing?					
